# Factors Influencing Depression Endpoints Research (FINDER): baseline results of Italian patients with depression

**DOI:** 10.1186/1744-859X-8-14

**Published:** 2009-05-29

**Authors:** Luigi Grassi, Andrea Rossi, Alessandra Barraco

**Affiliations:** 1 Section of Psychiatry, University of Ferrara, Italy; 2 Clinical Psychiatry Unit, Department of Mental Health, NHS Health Agency, Ferrara, Italy; 3 Medical Department, Eli Lilly Italia, Sesto Fiorentino, Italy

## Abstract

**Background:**

Factors Influencing Depression Endpoints Research (FINDER) is a 6-month, prospective, observational study carried out in 12 European countries aimed at investigating health-related quality of life (HRQoL) in outpatients receiving pharmacological treatment for a first or new depressive episode. Baseline characteristics of patients enrolled in Italy are presented.

**Methods:**

All treatment decisions were at the discretion of the investigator. Data were collected at baseline and after 3 and 6 months of treatment. Baseline evaluations included demographics, medical and psychiatric history, and medications used in the last 24 months and prescribed at enrolment. The Hospital Anxiety and Depression Scale (HADS), was adopted to evaluate depressive symptoms, while somatic and painful physical symptoms were assessed by using the Somatic Symptom Inventory (SSI) and a 0 to 100 mm visual analogue scale (VAS), HRQoL via 36-item Short Form Health Survey (SF-36), and the European Quality of Life 5-Dimensions (EQ-5D) instrument.

**Results:**

A total of 513 patients were recruited across 38 sites. The mean ± standard deviation (SD) age at first depressive episode was 38.7 ± 15.9 years, the mean duration of depression 10.6 ± 12.3 years. The most common psychiatric comorbidities in the previous 24 months were anxiety/panic (72.6%) and obsessive/compulsive disorders (13.4%), while 35.9% had functional somatic syndromes. Most patients (65.1%) reported pain from any cause. Monotherapy with selective serotonin reuptake inhibitors (SSRIs) and tricyclic antidepressants (TCAs) was prescribed at enrolment in 64.5% and 6.4% of the cases, respectively. The most commonly prescribed agents were sertraline (17.3%), escitalopram (16.2%), venlaflaxine (15.6%) and paroxetine (14.8%). The mean HADS subscores for depression and anxiety were 13.3 ± 4.2 and 12.2 ± 3.9, respectively; 76.4% of patients could be defined as being 'probable cases' for depression and 66.2% for anxiety. The mean total score of VAS-pain in the last week was 42.9 ± 27.1, with highest scores reported in the 'interference of pain with daily activities' and in 'amount of time patient was awake and had pain'. From SF-36, the worst health status was found for role limitations due to emotional problem, mental health and social functioning. A mean score < 50 (that is, below the standardised population norm) was also found in all remaining domains. The SF-36 summary scores and EQ-5D (health status and VAS) were lower in patients with moderate/severe pain than in those with no or mild pain.

**Conclusion:**

The baseline results of patients enrolled in the FINDER study in Italy show clinical and functional impairments, and poor HRQoL. The results obtained after 6 months of therapy will permit better understanding the effects of different variables on clinical outcomes and HRQoL.

## Background

Depression is a common and debilitating condition associated with significant mortality rates, whereby approximately 15% of patients who suffer from depression commit suicide [1]. Unipolar depression is currently ranked as the fourth major cause of disability worldwide [2], and is expected to rise to the second highest cause by 2020 [3]. Despite advances in the recognition and availability of new pharmacological therapies, a high rate of patients experience recurrence after a major depressive disorder when followed-up on a long-term basis [4], and it is estimated that no more than a third of depressed patients who begin treatment achieve full remission (that is, a complete relief of symptoms and return to full functioning in all areas of life) within 8 weeks of therapy [5].

A better understanding of the factors that influence the outcome of a depressive episode may help in patient management [6]. Comorbid anxiety disorders, demographic and social factors, and the presence of other medical diseases, were found to be associated with a negative outcome of a depressive episode and hence with a reduced possibility of achieving remission [7,8]. Taking into account that depression typically causes global effects on patient function, including psychological, physical, social and functional health state [9], the use of scales focused only on the mood symptoms may not provide a full understanding of factors influencing outcomes of depression. Therefore, the use of appropriate instruments for the assessment of health-related quality of life (HRQoL) covering a broader range of domains allows identification of those factors that have an impact on outcomes in a depressive episode in addition to the effects of treatment on the overall health state and on long-term prognosis.

The Factors Influencing Depression Endpoints Research (FINDER) study was a prospective, observational study designed to increase the understanding of the patient-related and disease-related factors that can influence outcomes, as measured by HRQoL scales, in patients clinically diagnosed with a depressive episode in the primary or specialist care setting and starting pharmacological treatment. The naturalistic design has been chosen to better reflect a real world setting, thus making the results generalisable to a wider population. The FINDER study was carried out in 12 European countries and allowed collection of not only pan-European data, but also of country-specific data.

The objective of this article is to examine the baseline results of patients from the FINDER study enrolled in 38 specialist sites in Italy and distributed throughout the entire national territory, and to assess the relationship between the characteristics of depression and impairment of HRQoL.

## Methods

### Study design

The FINDER study was a 6-month, prospective, observational, European study that included depressed patients starting pharmacological treatment in an outpatient setting. Adult patients where eligible for inclusion if they (1) presented within the normal course of care for depression, (2) required pharmacological treatment for either their first episode of depression or for a new episode, and (3) were not simultaneously participating in a different study that included an investigational drug or procedure. The participating physician, at his/her own discretion, had to decide to initiate pharmacological treatment for depression according to his/her own clinical practice. All treatment choices were at the discretion of the physician, according to the local standard of medical care. Patients were enrolled between May 2004 and September 2005.

The study design and baseline characteristics of the FINDER study population have been described in detail elsewhere [10], and so will be described here briefly. Data were recorded at baseline, and at follow-up visits carried out after 3 and 6 months after the start of therapy. The baseline evaluations included demographic data, medical and psychiatric history with comorbidities (including functional and somatic syndromes), and medications used in the last 24 months and prescribed at enrolment. In the present study, all the investigators participating in the 38 Italian centres of the FINDER were psychiatrists operating in a specialist setting and entered different numbers of patients depending on the size of their patient population.

### Instruments

Severity of depression and anxiety was measured using the Hospital Anxiety and Depression Scale (HADS) [11]. This is a 14-item self-assessment scale evaluating depression (7 items) and anxiety (7 items). Each item is answered by the patient in a 4-point (0 to 3 points) response category, to obtain a 0 to 21 score for both depression and anxiety. For each subscale a score of 0 to 7 can be regarded as a normal range, a score between 8 and 10 is indicative of a borderline case and scores > 11 are defined as 'probable caseness'.

Somatic and painful physical symptoms were assessed by using the Somatic Symptom Inventory (SSI) [12] and a 0 to 100 mm visual analogue scale (VAS). The SSI-28 is a self-report scale that assesses the degree to which various physical complaints had been bothersome to the patient. Each complaint is rated on a defined step scale from 1 (not at all) to 5 (a great deal), which consists of 21 non-painful and 7 painful somatic symptoms.

The VAS required the patients to evaluate their pain on a 0 to 100 scale across 6 different domains, which took into consideration pain in different parts of the body, the awareness of pain perception and the interference of pain with daily activities. A horizontal line ranging from 'no pain/interference' (0) to 'as severe as imaginable/complete disability' (100) was used in the assessment. A value > 30 was defined as a pain of at least moderate degree, according to a standard definition [13].

HRQoL was measured by using the Short Form-36 Health Survey (SF-36) and the European Quality of Life (EuroQoL) instrument. The SF-36 [14] consists of 36 questions covering 8 health domains (subscales): physical functioning, bodily pain, role limitations due to physical problems, role limitations due to emotional problems, general health perceptions, mental health, social function, and vitality. Each subscale is scored by summing the individual items and then transforming the scores into a 0 to 100 scale, with higher scores indicating better health status or functioning. The 2 summary scores, physical component summary (PCS) and the mental component summary (MCS), were obtained from the 8 SF-36 subscales [15] and transformed by using population-based scores with a mean of 50 (standard deviation of 10). Therefore, any value below 50 indicated worse than average scores.

The EuroQoL [16] is a measure of the health status index (HSI) that includes 5 (EQ-5D) dimensions of health (mobility, self-care, usual activities, pain/discomfort, anxiety/depression), each consisting of 3 levels (no, some/moderate and extreme problems), and a 0 to 100 VAS, with higher scores indicating better HRQoL.

### Data analysis

Descriptive summary statistics (means, standard deviations (SD), frequencies and percentages) were used to describe the baseline characteristics of the study population. Patients were excluded from the analysis if one or more entry criteria were violated or from individual analyses based on missing, implausible (according to predefined ranges) or un-interpretable data. Data were analysed using SAS Software (version 8.2; SAS Institute, Cary, North Carolina, USA).

## Results

### Demographic data and psychiatric history

A total of 513 patients were recruited in 38 specialist sites in Italy. Demographic characteristics of patients are shown in Table 1. The mean ± SD age was 49.2 ± 15.2 years and females were predominant (72.9% of total sample). Approximately half of patients were married and 34.4% were employed.

**Table 1:** Demographic data and patients' baseline characteristics

Characteristic	Results
Demographic data:	
Age, years, mean ± SD (range)	49.2 ± 15.2 (18 to 85)
Gender, M/F, N (%)	M: 139 (27.1); F: 373 (72.9)
BMI, kg/m^2^, mean ± SD (range)	25.4 ± 4.9 (16.0 to 72.3)
Educational level:	
Mandatory or no education, N (%)	302 (58.9)
Further education, N (%)	211 (41.1)
Family status:	
Married, N (%)	290 (56.6)
Divorced, N (%)	27 (5.3)
Legally separated, N (%)	32 (6.3)
Widowed, N (%)	47 (9.2)
Domestic partner, N (%)	19 (3.7)
Partner, living separate, N (%)	26 (5.1)
No relationship, N (%)	71 (13.9)
Occupational status:	
Employed, N (%)	176 (34.4)
Unemployed, N (%)	63 (12.3)
Retired, N (%)	136 (26.6)
Volunteer work, N (%)	4 (0.8)
Full-time housewife, N (%)	111 (21.7)
Student, N (%)	22 (4.3)
Smokers, N (%)	152 (29.7)

BMI, body mass index; SD, standard deviation.

Table 2 shows the patients' psychiatric history and comorbidities. The mean duration of depression was 10.6 ± 12.3 years and the mean age at the first depressive episode was 38.7 ± 15.9 years. The mean duration of the current episode was 12.8 ± 14.5 weeks, and, in patients with previous depressive episodes in the last 24 months (N = 264, 51.7% of total sample), a mean time of 22.4 ± 17.4 weeks had elapsed between the remission of the last and the start of the current episode. Anxiety/panic disorders were the most common psychiatric comorbidity in the last 24 months and were reported in 72.6% of patients. Functional somatic syndromes were reported in 35.9% of patients: chronic fatigue syndrome and irritable bowel syndrome were the most common syndromes (in 17.1% and 16.6% of cases, respectively). A rate of 65.1% had pain at enrolment: a defined medical disorder known to cause pain was present in 20.3% of patients. A physical trauma in the past 24 months that caused pain present at enrolment was reported in 37 (7.3%) patients. A rate of 47.8% of patients suffered from one or more of a prespecified list of other non-psychiatric diseases: arterial hypertension (25.4%) and rheumatological disorders (16.2%) were the most common conditions.

**Table 2:** Psychiatric history and comorbidities/functional syndromes

Parameter	Value
Psychiatric history at enrolment, mean ± SD (range):	
Duration of depression, years	10.6 ± 6.0 (0 to 63)
Age at first major depressive episode, years	38.7 ± 15.9 (14 to 82)
Duration of the current major depressive episode, weeks	12.8 ± 14.5 (1 to 100)
No. of previous depressive episodes in the last 24 months	1.9 ± 1.1 (1 to 10)
Duration of the last major depressive episode*, weeks	14.3 ± 11.5 (1 to 60)
N (%) of patients with other psychiatric comorbidities in the last 24 months:	
Anxiety/panic disorders	371 (72.6)
Obsessive-compulsive disorders	68 (13.4)
Bipolar disorders	20 (3.9)
Schizophrenia	4 (0.8)
Drug/alcohol dependence disorders	27 (5.3)
Any of these psychiatric disorders	394 (77.3)
N (%) of patients with functional syndromes at enrolment:	
Irritable bowel syndrome	84 (16.6)
Chronic fatigue syndrome	86 (17.1)
Atypical chest pain	57 (11.2)
Irritable bladder	46 (9.1)
Fibromyalgia	50 (9.9)
Chronic pelvic pain	22 (4.3)
Any of these functional syndromes	182 (35.9)
Pain summary at enrolment, N (%) of patients:	
No/mild pain	167 (34.9)
Significant pain with medical disorder known to cause pain	97 (20.3)
Significant pain with medical disorder not associated with pain or without comorbidity	215 (44.9)

*In patients with one or more previous episode.

SD, standard deviation.

### Medications taken before and prescribed at baseline

A total of 40.5% of patients received a medication for depression in the last 24 months (monotherapy with selective serotonin reuptake inhibitor (SSRI) was predominant, taken in 20.3% of patients), while 19.2% additionally received psychotherapy and 68.8% an analgesic medication in the same time period.

Monotherapy with SSRIs and tricyclic antidepressants (TCAs) was prescribed at baseline in 331 (64.5%) and 33 (6.4%) patients, respectively; other single agents were given in 120 patients (23.4%) and combined therapy in 29 (5.7%). A single antidepressant agent was prescribed at enrolment in most of patients (93.8%). The antidepressants prescribed in at least 10% of patients were: sertraline (89 patients, 17.3%), escitalopram (83, 16.2%), venlafaxine (80, 15.6%), paroxetine (76, 14.8%) and citalopram (62, 12.1%).

### Clinical health status

The mean HADS subscores for depression and anxiety were 13.3 ± 4.2 (range 1 to 21) and 12.2 ± 3.9 (range 0 to 21), respectively. 'Probable cases' (that is, a score > 11 on each HADS subscale) were 76.4% for depression and 66.2% for anxiety; borderline cases (that is, a score of 8 to 10) were 14.8% for depression and 20.9% for anxiety; and non-cases (that is, a score of 0 to 7) were 8.8% for depression and 12.9% for anxiety. There was no clear relationship between mean HADS subscores for depression and anxiety and the duration of the current depressive episode. Conversely, the mean scores for both HADS subscales were lower (that is, indicative of a better state) in patients with no or mild pain compared to those with painful conditions, irrespective of the presence of medical disorders known to cause pain (Table 3). Lower mean values in both subscales were found in patients without psychiatric comorbidities than in those with concomitant psychopathologies.

**Table 3:** Results of HRQoL and clinical health status in presence/absence of pain: values are mean scores ± standard deviation (SD)

Parameter	No/mild pain	Significant pain with medical disorder known to cause pain	Significant pain with medical disorder not associated with pain or without comorbidity
SF-36 PCS	50.0 ± 9.1	37.5 ± 7.8	43.4 ± 7.9
SF-36 MCS	24.1 ± 10.3	22.0 ± 6.8	20.7 ± 8.9
EQ-5D	0.53 ± 0.28	0.27 ± 0.34	0.37 ± 0.30
EQ-VAS	51.4 ± 19.5	41.3 ± 18.1	42.0 ± 19.0
HADS depression	12.0 ± 4.4	13.8 ± 3.8	13.8 ± 4.1
HADS anxiety	10.6 ± 3.8	12.9 ± 3.5	13.0 ± 3.9

EQ-5D: EuroQoL 5 dimensions; EQ-VAS: EuroQoL visual analogue scale; EuroQoL, European Quality of Life instrument; HADS: Hospital Anxiety and Depression Scale; SF-36 MCS: Short Form-36 mental component summary; SF-36 PCS: Short Form-36 physical component summary.

**Figure 1:**
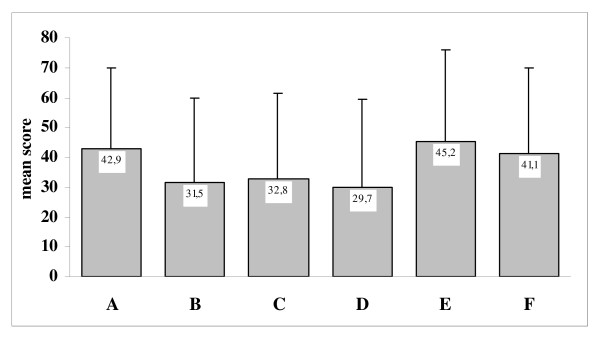
**Results of self-assessment of pain on 0 to 100 visual analogue scale (VAS): values are means, with standard deviations in bars**. (a) Overall pain score; (b) severity of headaches during the last week; (c) severity of back pain during the last week; (d) severity of shoulder pain during the last week; (e) interference of overall pain with ability to do daily activities during the past week; (f) time with pain while awake.

The mean scores on SSI-28 were 2.4 ± 0.7 for all items, 2.3 ± 0.8 for pain items and 2.4 ± 0.7 for somatic items. Figure 1 shows the results of the self-assessment of pain measured by the 0 to 100 VAS. The mean overall score of VAS related to pain symptoms in the last week was 42.9 ± 27.1 (median 45.0), with highest scores reported in the interference of pain with daily activities (mean 45.2) and in amount of time the patient was awake and had pain (mean 41.1).

### Health-related quality of life

**Figure 2:**
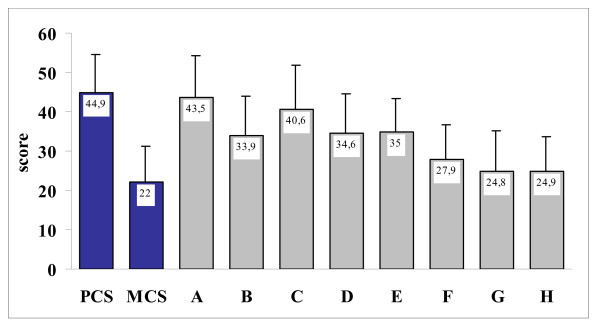
**Results of Short-Form-36 Health Survey (SF-36) physical component summary (PCS), mental component summary (MCS) and domains; values are means, with standard deviations in bars**. (a) Physical functioning; (b) role limitations due to physical problems; (c) bodily pain; (d) general health perception; (e) vitality; (f) social functioning; (g) role limitations due to emotional problems; (h) mental health.

The results of SF-36 are shown in Figure 2. The mean PCS and MCS scores were 44.9 ± 9.7 and 22.0 ± 9.2, respectively. Among all patients, the worst health status was found for role limitations due to emotional problems (24.8 ± 10.3), mental health (24.9 ± 8.6) and social functioning (27.9 ± 8.8). A mean score less than 50 (that is, below the population norm) was also found in all the other domains.

**Figure 3:**
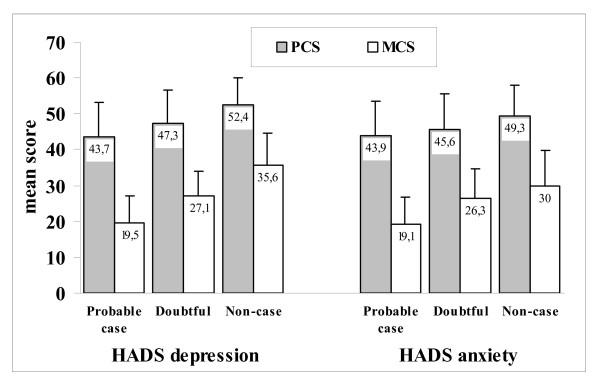
**Results of physical component summary (PCS, grey columns) and mental component summary (MCS, white columns) of the Short-Form 36 Health Survey (SF-36) by Hospital Anxiety and Depression Scale (HADS) caseness of anxiety and depression**. Values are means, with standard deviations in bars.

The results of SF-36 PCS and MCS by HADS caseness of anxiety and depression are shown in Figure 3. Cases of both anxiety and depression were associated with the lowest mean scores for both PCS and MCS (that is, with the worst HRQoL perception). There was no relationship between the PCS and MCS mean scores and the presence/absence of a previous episode of depression in the last 24 months, and there was no clear relationship between PCS/MCS mean scores and the duration of the current depressive episode.

The mean score of the EQ-5D HSI in the total population was 0.40 ± 0.31, while the mean score of VAS was 45.7 ± 19.6. Again, there were no differences in EQ-5D HSI and VAS mean scores between patients with or without episodes in the last 24 months, and the mean scores did not depend on the duration of the current depressive episode.

The mean scores for both SF-36 subscales and for EQ-5D (health state index and VAS) were lower in patients with significant pain (with or without medical disorders known to cause pain) than in those with no or mild pain (Table 3). The results for the SF-36 did not vary in patients with or without previous psychiatric comorbidities, whereas the mean scores for EQ-5D and VAS indicated a better level of HRQoL in patients without psychiatric comorbidities compared to those with any other previous psychopathology. EQ-5D HSI and VAS were also worse in patients with concomitant non-psychiatric disease than in patients with no other chronic medical conditions.

## Discussion

Depressive episodes are associated with substantial worsening of HRQoL and account for a greater impairment on all domains of commonly used HRQoL measures to that reported with non-psychiatric medical disorders [17]. Therefore, the use of multidimensional HRQoL scales that take into account a broad range of domains may help in understanding which factors influence depression outcomes and to which extent the coexisting psychiatric and non-psychiatric disease influence global functioning and subjective well-being.

This study examined the characteristics of patients with depression enrolled in outpatient psychiatric services in Italy and the relationship between the characteristics of depression (for example, severity and chronicity of depression, anxiety symptoms, painful symptoms), the impairment of HRQoL and the treatment patterns.

Consistent with the results obtained in the entire European population [18], the baseline results of the patients enrolled in the Italian sites have shown that functional somatic syndromes were reported in 35.9% of patients.

The mean HADS subscores for depression and anxiety in patients enrolled in Italy were 13.3 and 12.2, respectively, indicating a high comorbidity between depression and anxiety in patients being treated with antidepressants.

A rate of 65.1% of patients enrolled in the Italian sites had pain at enrolment according with Kelly definition, although a defined medical disorder known to cause pain was present in only 20.3% of the patients examined at baseline. The relatively high rate of patients reporting pain in absence of a recognised medical disorder associated with pain or without further comorbidities might be explained on the basis of the well-known correlation between medically unexplained symptoms and comorbid anxiety and depression disorders [19,20], as well as on the presence of multiple otherwise unexplained symptoms in somatic functional disorders, all of which share a psychopathologic causative origin [21].

The results for baseline SF-36 domains in the Italian patients were similar to those obtained in the overall European population in all examined parameters [10], including mean scores for PCS and MCS, mean scores for individual domains, type of domains showing the worst health status (which were limitations due to emotional problems, mental health and social functioning). Moreover, all examined domains had a mean score < 50 (that is, below the population norm) both in Italy and in all European countries.

In our sample of patients, pain was shown to affect HRQoL perception, both measured by means of the two SF-36 subscales (mental and physical components) and of EQ-5D (health state and VAS), as well as the level of depression and anxiety measured by means of the two HADS subscales was affected by concomitant pain. The median total score for overall pain VAS related to symptoms in the last week was 45.0, thus indicating that a relevant amount of patients were suffering of significant pain (that is, with a score in overall pain VAS ≥ 30). The results of the SSI-28 also showed no differences in mean scores between the pain and somatic items. As in the European population [10], the 'interference of pain with daily activities' and the 'amount of time patient was awake and had pain' were the two items of the pain VAS scale that showed the highest level of impairment.

The concomitant presence of both psychiatric and non-psychiatric diseases was associated with a worse EQ-5D health status and VAS mean scores. Conversely, the presence/absence of a previous episode of depression in the last 24 months and the duration of the current depressive episode were factors that did not influence both the mean scores for the mental and physical components of the SF-36 and the mean HADS subscores for depression and anxiety.

The results of the baseline data collected in the overall European population [10] have shown that, consistently with findings of a recent review of epidemiological studies conducted in Europe [22], more than 40% of patients had at least one comorbid chronic medical condition. Patients without any comorbidity showed a better outcome on all domains of the physical component of the SF-36, while depression was shown to have considerable influence on the mental summary of the SF-36. Both health state and VAS of the EQ-5D scores were less than half of the maximum possible score and were further reduced in patients with comorbidities. Further analysis of the Italian subsample will add information about the practice of Italian psychiatrists in the management of depression and in the attention to the somatic components/comorbidities of depression.

Certain limitations of the study should be mentioned. First, the results presented here derive from a series of centres not necessarily representing all the several types of psychiatric outpatient services in the country. A second limitation is that, even though the study is an observational one, criteria for recruitment did not request a formal psychiatric diagnosis of depression according to the classification systems usually employed (for example, the Diagnostic and Statistical Manual of Mental Disorders, 4th edition text revision (DSM-IV) and International Classification of Diseases, 10th edition (ICD-10)).

In summary, the baseline data derived from the Italian cohort of the FINDER study provide important information on the level of clinical and functional impairment, and of worsening of HRQoL, in patients with depression usually seen in outpatient psychiatric services for receiving pharmacological treatment. Furthermore, the role of several factors in worsening the patients' HRQoL perception at enrolment can be of clinical relevance in daily practice. The longitudinal data of the patients will give country-specific information on those factors that may influence the patients' HRQoL outcomes after a 6-month treatment for depression has been administered. In this respect, the FINDER study was designed with no restrictive limitation in terms of prescribed pharmacological treatment, which was left at the complete discretion of the treating psychiatrist, in order to have a real-life sample of patients initiating treatment for a depressive episode. The contribution of depression and comorbidities, as well as pain perception, will be taken into account when interpreting changes in SF-36 and EQ-5D scores following antidepressant treatment.

## Competing interests

This study was sponsored by Eli Lilly and company. AR and AB are currently working for Eli Lilly.

According to Italian law, honoraria for recruiting patients were paid to participating Institutions.

## Authors' contributions

AR and AB made substantial contributions to analysis and interpretation of data, and in the revision of the manuscript. LG gave relevant contribution in the recruitment of patients, in the critical revision of the manuscript, and in final approval prior to publication.
